# Challenges in Simulating Light-Induced Processes in DNA

**DOI:** 10.3390/molecules22010049

**Published:** 2016-12-29

**Authors:** Philipp Marquetand, Juan J. Nogueira, Sebastian Mai, Felix Plasser, Leticia González

**Affiliations:** Institute of Theoretical Chemistry, Faculty of Chemistry, University of Vienna, Währinger Straße 17, 1090 Vienna, Austria; philipp.marquetand@univie.ac.at (P.M.); nogueira.perez.juanjose@univie.ac.at (J.J.N.); sebastian.mai@univie.ac.at (S.M.); felix.plasser@univie.ac.at (F.P.)

**Keywords:** DNA, photochemistry, excited states, simulation, electronic structure, ab initio molecular dynamics, QM/MM, theoretical chemistry

## Abstract

In this contribution, we give a perspective on the main challenges in performing theoretical simulations of photoinduced phenomena within DNA and its molecular building blocks. We distinguish the different tasks that should be involved in the simulation of a complete DNA strand subject to UV irradiation: (i) stationary quantum chemical computations; (ii) the explicit description of the initial excitation of DNA with light; (iii) modeling the nonadiabatic excited state dynamics; (iv) simulation of the detected experimental observable; and (v) the subsequent analysis of the respective results. We succinctly describe the methods that are currently employed in each of these steps. While for each of them, there are different approaches with different degrees of accuracy, no feasible method exists to tackle all problems at once. Depending on the technique or combination of several ones, it can be problematic to describe the stacking of nucleobases, bond breaking and formation, quantum interferences and tunneling or even simply to characterize the involved wavefunctions. It is therefore argued that more method development and/or the combination of different techniques are urgently required. It is essential also to exercise these new developments in further studies on DNA and subsystems thereof, ideally comprising simulations of all of the different components that occur in the corresponding experiments.

## 1. Introduction

The study of the photochemistry of DNA has become a trend in photobiology, attracting researchers from different fields [1,2,3,4,5,6,7]. Understanding the interaction of UV light with DNA and its building blocks is important because it can provide answers to questions like “Which damage can light produce in our genetic code?” or “Which is the role of photostability in prebiotic chemistry?”. The primary response of DNA to light is ultrafast and occurs on time scales as small as femtoseconds (10${}^{-15}$ s). Among the physical processes taking place after light irradiation are intramolecular vibrational relaxation, internal conversion through conical intersections and intersystem crossing between states of different multiplicity. These process are accompanied by particular molecular deformations, which in turn determine the fate of the involved electronic states and which might affect the general structure of DNA and eventually its biological function or that of its components. From the theoretical point of view, the key step in understanding the photophysics and photochemistry of DNA is to get a picture of the time-dependent population of the electronic states after light irradiation [3,4,5,6]. Further steps include, e.g., the generalization of findings for single building blocks to rules for a whole class of structures [8,9] or the prediction of reaction mechanisms [10].

Most of the photophysical and photochemical events are difficult to observe experimentally [3,4,5,6], not only because they are ultrafast or because some species are only metastable, but most importantly because experiments observe population dynamics only indirectly. It could be thought as looking at an image through a lens, and it is hard to know whether the lens has distorted the original image or not. Oftentimes, different experimental techniques seem to deliver different results, which might be only different pieces of the same puzzle. However, even when different experimental techniques yield, for instance, the same time scales, the help of theory is needed in most cases to interpret the results. Theoretical simulations of the response of DNA to light are invaluable to rationalize the experimental results and put the different parts of the puzzle together. However, the theoretical modeling of the DNA photochemistry and photophysics is not straightforward either. To model the same observable as measured in experiments, theoreticians are confronted with a large assortment of techniques that can be applied to each of the different processes involved in that experiment. Unfortunately, each of these methods has pitfalls and can lead to different results. Thus, it is not surprising to find frequently different interpretations of the same experiment, to the despair of the experimentalist.

A well-known source of conflict in DNA simulations is the choice of the level of electronic structure theory to characterize the ground and excited state potential energy surfaces, as well as associated electronic properties. For instance, the correct depiction of DNA interactions can be quite defiant, since it involves describing bond formation and breaking correctly, among other challenges. Many methods, including the widely-used density functional theory (DFT), have problems in providing accurate potential energies for these processes [11,12,13]. Further challenges for electronic structure calculations in DNA are the correct description of hydrogen bonds [14], as well as stacking and dispersion interactions [15,16,17]. Formally easy, but difficult in practice is also to distinguish between local and non-local excitations, differentiate states with multiple-excitation character [10], identify the amount of charge transfer and quantify excitons [18].

An obvious component in the simulation of photoinduced processes is modeling explicitly the initial excitation of DNA or its nucleobases, as it happens in the experiment. Yet, this explicit interaction is often neglected in the simulations. When it is not, the description of light-matter interactions relies inevitably on approximations. The most prominent one is probably the dipole approximation, where the wavelength of the light is assumed to be much larger than the considered molecule, and thus, the spatial modulation of the electromagnetic field is neglected. For larger DNA strands and also for strong fields [19], this approximation is not valid. However, we are not aware of any study tackling the formidable task of treating explicitly light interaction with DNA beyond the dipole approximation. Another challenge related to this step is the modeling of multi-photon processes [20], as they occur in the time-resolved experiments or the simulation of DNA processes, where the focus lies on the electron dynamics, such as involving ionization [21,22]. The same limitations of theory apply for any subsequent interactions with light, e.g., for the simulation of a probe process, where the latter is an essential ingredient of the experiment.

The calculation of the actual nuclear photoinduced dynamics is probably the part of the simulation that currently faces the most challenges. Ideally, one should be able to describe within the dynamics all kinds of quantum effects, such as tunneling [23], interference effects [24] or decoherence [25,26], that can arise in the extended molecular systems that constitute the DNA. Such effects can only be fully included when using exact quantum dynamics methods, but as it will be outlined later, such a methodology is out of the question for systems of the size of DNA or even some of its smaller building blocks. A number of alternatives, as described below, arrive at the desired accuracy.

Last, but not least, the analysis of the computational results can also come with its own challenges. One usual problem in dynamics simulations is to keep track of the electronic evolution of the wavefunction in time, e.g., to follow the character of the involved orbitals [27,28,29]. In addition, it is not always easy to identify the main pathway in the nuclear evolution, and thus, sophisticated analyses of internal coordinates and reaction pathways may be necessary [30]. In this sense, often the mere amount of data to process can already be daunting. The simulation of large systems such as DNA strands easily produces results that deserve the term “big data” and cannot be processed in a manual way.

This long and yet non-exhaustive list of challenges related to the simulation of excited-state dynamics in DNA vividly demonstrates how demanding such computations are and shows that there is room for improvement. In the following, we will give an overview of the most commonly-used approximations employed in each of these tasks and envision the corresponding technical approaches that ideally should be employed. Note that the following discussion will focus on processes induced by direct excitation of DNA or its constituents, and thus, topics like, e.g., DNA repair, oxidative DNA damage or DNA photosensitization are not included.

## 2. Potential Energy Surfaces

The first step in the simulation of electronically-excited states of DNA components is the choice of a suitable electronic structure method to deliver accurate potential energies. Already, the computation of the vertical excitation energies of individual nucleobases is challenging, as excited states of different characters (e.g., $\pi \pi^{*}$, $n\pi^{*}$, Rydberg) have to be taken into account, and a balanced description of these states has to be assured. However, it is not just enough to describe these states correctly in the Franck–Condon region; it is necessary to provide a correct description of the potential energy surfaces, as well as their intersections, over the whole range of nuclear geometries accessed during the dynamics simulations. When several nucleobases interact, a number of new phenomena, such as charge transfer states, excitons, exciplex formation and dimerization, can come into play. Realistic simulations of these processes require the simultaneous quantum mechanical description of several DNA bases and ideally the inclusion of the backbone and solvent through environmental models.

A hierarchy of different methods is available to describe the excited states and potential energy surfaces of interacting nucleobases [31]. A starting point is given through the usage of effective exciton models [32,33,34] or the application of semi-empirical models, either based on wavefunctions [35,36,37,38] or on density functional tight binding (DFTB) [39,40]. Such approaches allow for an efficient description of large systems and extended dynamics simulations. However, a careful parameterization is required for their successful application, and in case such a parameterization is unavailable, one can resort to ab initio methods. The latter are to a large extent the choice of many key players in the field. Time-dependent density functional theory (TDDFT) is widely applied to calculate excited states of DNA fragments [41,42,43,44,45]. The main challenge in this case is the choice of an appropriate functional, where in particular, the fraction of Hartree–Fock exchange has a crucial impact on the resulting excitation energies [41,44,45]. The next step on the ladder is given by wavefunction-based methods—for example correlated second-order methods, such as approximate coupled cluster (CC2), algebraic diagrammatic construction (ADC) or perturbatively-corrected configuration interaction with single excitations (e.g., CIS(D))—which can be successfully applied [18,46,47,48,49]. Furthermore, more involved coupled cluster methods have been used to compute the excited states of DNA components [50].

In order to describe the potential energy surfaces correctly in all regions, including intersections between the ground and excited states (which is particularly important for dynamics), it is necessary to move to a multireference description. Complete active space self-consistent field (CASSCF) is a good starting point, providing at least a qualitatively correct description of the multireference effects of interest, and has indeed been widely applied in studies of DNA constituents [10,51,52,53,54,55,56,57]. A more accurate quantitative description of the excited states is obtained by adding dynamic correlation on top of a CASSCF reference. A very popular approach amounts to including dynamic correlation perturbatively through second-order perturbation theory on the CASSCF wavefunctions, which is known as CASPT2 [16,58,59,60,61,62]. Multireference configuration interaction (MRCI) offers an alternative [53,63,64,65]. The availability of analytical gradients and nonadiabatic couplings makes this method suitable for the optimization of conical intersections [63] and also for dynamics studies [9,66].

It should be realized that the choice of the proper electronic structure method for computations on DNA is a very delicate task, and much care has to be exercised to interpret the results. Taking two studies on adenine as an illustration [58,67], it was found that CASSCF and TDDFT using the M052X functional overestimated the energy of the La1 state by about 2 eV and 0.8 eV, respectively. Correlated ab initio methods, such as CC2, ADC(2) and CASPT2, tend to provide more reliable excitation energies, but a series of detailed coupled cluster benchmark studies concluded that not even these methods are capable of describing all aspects of the excitation spectra [50]. If a quantum chemical method is chosen as the basis for a dynamical description, new challenges come into play, as even strongly-distorted structures have to be described properly. For that purpose, multireference methods are generally best suited and thus usually applied [9,10,51,52,54,55,57,66]. In the example of adenine, it was found that ADC(2) also showed a proper description of the ultrafast deactivation, while TDDFT using various functionals failed [68]. When interacting bases are considered, charge transfer states come into play, which are problematic for TDDFT [41]. A particularly challenging task is the description of processes where the nucleobases approach each other, such as exciplex formation [18,38,67,69] and dimerization [10,70,71], considering that the evaluation of accurate interaction potentials between nucleobases is already difficult in the ground state [15]. Aside from the general description of the excited states, it is a particular problem that, when the nucleobases approach each other, there is a strong basis set superposition error [59] that cannot be remedied by a simple counterpoise correction [69].

Besides the choice of an appropriate electronic structure method, the description of the nucleic acid environment is another crucial factor that influences the calculation of potential energy surfaces. Without a doubt, the calculation of the properties of individual nucleobases in the gas phase and in solution has provided invaluable insight [5,6]. However, the inclusion of the biological environment is decisive to obtain a realistic picture of the photophysics and photochemistry of DNA strands. Nowadays, computational resources and appropriate methodologies are, in principle, available to go beyond the monomeric level and can be pushed to simulate multi-chromophore systems, including environmental effects. As such, the next logical step after the study of isolated nucleobase monomers is to analyze their excited states embedded into a solvated DNA strand. The few nonadiabatic surface hopping simulations carried out in this spirit have shown that electrostatic interactions with neighboring nucleobases and Watson–Crick hydrogen bonding have a strong influence, e.g., on the excited-state decay of adenine [72,73] and guanine [14], slowing down their decay to the ground state. Thus, it is clear that the conclusions drawn from simulations of isolated nucleobases, while appropriate to explain gas phase experiments [74], cannot be directly extrapolated to the macromolecular level.

Usually, the effect of the environment is taken into account by a hybrid quantum mechanics/ molecular mechanics (QM/MM) approach [75], in which the nucleobase of interest is described quantum mechanically (QM), and the rest of the system is treated by molecular mechanics (MM) using a force field. The interaction between both layers is often calculated by an electrostatic embedding, in which the MM charges are included in the Hamiltonian of the QM region. Thus, the environment polarizes the electronic density of the QM region, but the QM region does not affect the electronic structure of the MM subsystem due to the fixed-charge nature of the force fields usually employed [76,77,78,79]. This is a reasonable approach if the electronic structure of the QM subsystem does not suffer drastic changes and therefore does not introduce important alterations in the MM region. If, however, light absorption causes a strong electronic redistribution in the QM region, the MM atoms located close to the QM region should respond accordingly. Such a response can be achieved by using a QM/MM polarizable embedding [75] in combination with polarizable force fields, enabling a mutual polarization between the QM and MM subsystems. Two prominent examples are the MMpol [80] and CHARMM Drude [81] force fields, which include induced dipole moment contributions that can respond to changes in the polarity of the environment. Although polarizable formulations are clearly superior to electrostatic schemes, their use in excited-state calculations is still limited because of their high computational cost, especially if mutual polarization between the QM and MM layers is considered in a self-consistent manner. A common feature of the different QM/MM formulations is that they describe properly only the electrostatic interactions between the two layers, while the exchange repulsion interactions are neglected or crudely considered by the repulsive terms of Lennard–Jones potentials. The wrong description of exchange repulsion can cause a nonphysical penetration of the electronic density of the QM region into the MM subsystem if, for example, the excited states of the QM region are rather diffuse. More accurate hybrid QM/QM’-based techniques, which combine two levels of QM theory and can be complemented with a third MM layer, such as polarizable density embedding [82] and frozen density embedding [83], have been developed to overcome this problem. However, their computational cost is considerably higher than the one of the electrostatic or polarizable QM/MM methods, and their use is sparse in the simulation of excited-state processes of nucleobases and DNA fragments.

Despite the remarkable success of QM/MM and QM/QM’(/MM) approaches, they can describe the environment only as a secondary actor in the main process under study, in which the environment can induce alterations in the topology of the QM potential energy surfaces. However, if the environment (e.g., neighboring nucleobases or solvent molecules) plays an active role in the process, the relevant part of the environment needs to be included in the QM region. Such a procedure was followed, for example, when studying the exciton and charge-transfer character of excited states of oligonucleotides [48], photoinduced cyclobutane thymine dimerization [10,84] or excited-state relaxation of 7H-adenine mediated by an electron transfer from the solvent [85]. However, the expansion of the QM subsystem is accompanied by a considerable increase in computational cost. Thus, the progress of simulating DNA excited-state processes clearly depends on the development of computationally-efficient electronic structure methods.

The path to the solution of the above-mentioned problems will be supported by the newest developments in electronic structure theory and multiscale methods. Modern highly accurate, but yet efficient, ab initio methods, such as the density matrix renormalization group (DMRG) [86,87,88] or full configuration interaction quantum Monte Carlo (FCIQMC) [89], can provide new high-end reference computations. The accessible system sizes can be expanded using new ideas in the implementation of electronic structure methods, e.g., through density fitting [90,91], through linear-scaling methods [92], through parallel computing [93] or through quantum chemistry codes optimized for graphical processing units [94]. A further step in the direction of larger system sizes is the application of fragment-based multiscale models, i.e., models that require quantum chemistry computations only on individual molecules. So far, fragment-based models have been applied to charge transport in DNA using a DFTB/MM framework [95] and to excited states in photosynthetic complexes using TDDFT [96], and an extension to DNA excited states is certainly feasible. Another exciting approach is the use of machine learning, where larger systems could in principle be described with QM accuracy, but at the speed of classical force fields [97,98,99,100,101,102].

## 3. Excitation Process

The starting point for a realistic dynamics simulation in the excited state is to obtain a reasonable set of initial conditions, i.e., a ground state wavefunction or the corresponding geometries and velocities. The two most employed approaches to sample the initial conditions in a classical framework are the quantum sampling and thermal sampling methods [103]. In the first approach, the coordinates and velocities of each normal mode are chosen based on a harmonic-oscillator Wigner distribution, in which the zero-point vibrational energy is given to each normal mode [104]. In the thermal sampling, initial conditions are selected from a ground-state molecular dynamics simulation at a given temperature, in which the energy of each normal mode is kT. At first glance, the quantum sampling is the best way to generate initial conditions because the chromophore receives the quantum mechanical vibrational energy. Accordingly, it is the preferred approach for most of the dynamics simulations performed in the gas phase [62,105,106]. However, because quantum sampling relies on the harmonic oscillator approximation, normal modes with high anharmonicity, generally low-frequency vibrations, are badly described. Therefore, if low-frequency motions play a relevant role in the photophysics of the chromophore, the use of harmonic quantum sampling can lead to the wrong behavior in the dynamics. In contrast, thermal sampling can properly describe anharmonic motions, especially if the potential energy is computed with a quantum mechanical method (ab initio molecular dynamics). However, the vibrational energy is underestimated, and high temperatures are required to properly explore the configurational space [103]. Thermal sampling is usually employed for generating initial conditions in the condensed phase [84,107,108] because the frequency calculation required for quantum sampling is unfeasible for large system sizes. Moreover, the harmonic approximation in the presence of an environment is generally worse than in the gas phase calculations due to the large amount of low-frequency intermolecular normal modes. A combination of both sampling methods has been used for simulations in the condensed phase [109,110]. In such a hybrid approach, quantum sampling is performed for the chromophore in the gas phase, and the configurational space of the environment is sampled by molecular dynamics with the chromophore constrained at the quantum geometries. It is not clear whether this approach is better than a full thermal sampling because in the hybrid approach, the environment does not have any effect on the geometries and velocities of the chromophore, which are generated in the gas phase. Moreover, the fact that the vibrational energy of the chromophore is much higher than the one of the solvent generates a hot region in the system that can introduce artifacts in the subsequent dynamics simulation. Thus, further research is necessary to benchmark the current practices for the generation of initial conditions and to develop more appropriate approaches, especially in the condensed phase. Conformational motions of DNA single and double strands involve the participation of a large amount of vibrational normal modes, including low-frequency backbone and inter-base vibrations. Thus, the generation of initial conditions for electronic excited-state simulations of nucleobases or DNA fragments should ideally be based on anharmonic models that include the zero-point vibrational energy, at least for the relevant vibrational normal modes.

Once a suitable distribution in the electronic ground state has been obtained, the explicit excitation to one or several excited states has to be modeled. While different possibilities to excite a molecule exist, here, we restrict ourselves to excitation with electromagnetic radiation. The most realistic way to simulate the excitation process is to directly model the time-dependent laser-matter interaction, as taking place in ultrafast laser experiments. How a time-dependent electromagnetic field is incorporated technically in a calculation depends on the method used for the dynamics; see, e.g., [111,112,113,114]. Note that in surface-hopping simulations, optimally, one should use the Floquet picture for the laser-matter interaction [115,116,117].

Many studies use only an approximative treatment of the excitation process. A crude approximation is to directly project the ground-state distribution to the excited state. Such an approach is also called Δ-pulse excitation (a pulse that is infinitely short, i.e., comprises all energies) and uses in its simplest form the Condon approximation, i.e., assumes that the transition dipole moment, which couples the electric field and the molecule, is constant and equal to one for every geometrical configuration. An improved variant of the Δ-pulse excitation employs oscillator strengths as a selection criterion for population transfer to a specific excited state [118].

Trajectory-based methods often rely on such a simple Δ-pulse approach. The reason is that most laser pulses excite only a tiny fraction of the overall ground-state population, and a time-dependent description of the laser excitation would therefore use the vast majority of computational power to compute equilibrium ground-state dynamics in order to get a statistically meaningful number of trajectories transferred to the excited states. Therefore, it is preferable and computationally less expensive to carry out the excited-state dynamics selectively without running trajectories in the ground state by using the Δ-pulse variant for the excitation.

In general, however, it is desirable to describe the light-matter interaction explicitly in order to adequately model experiments. In such cases, i.e., when a time-dependent description of the excitation process is carried out, different approximations are usually made. For example, only the electric component of the electromagnetic field is commonly taken into account; the spatial variation of the field is neglected (dipole approximation); and also the dipole moments are only calculated as first-order terms, while higher order terms like the polarizability and the hyperpolarizabilities are often neglected. The different approximations may break down for strong fields [19], short wavelengths (e.g., X-ray radiation) [119] or large molecular systems, such as complete DNA strands. Despite these challenges, the simulation of light-matter interactions is in principle possible, and it should ideally be carried out explicitly.

## 4. Nuclear Dynamics

Arguably, the central step in the study of the excited-state dynamics of DNA fragments is the simulation of the actual nuclear motion after photoexcitation to the different potential energy surfaces. Only through these simulations, it is possible to obtain a full interpretation of the excited-state processes triggered experimentally, including the possibility to predict excited-state lifetimes, branching ratios between different reaction channels and to obtain all of the details of the nuclear rearrangements along these channels in real time. The latter is especially important to follow the ultrafast processes, which are commonly found in DNA fragments.

The main challenge in simulating nuclear motion is the description of intrinsic quantum-mechanical effects, for example tunneling [23], coherence [25,26], interference [24], zero-point energy or delocalization. The most straightforward, and most rigorous, approach to describe nuclear dynamics is therefore to numerically integrate the time-dependent Schrödinger equation, with a nuclear wave packet often expressed through some suitable grid-based basis functions. This so-called wave packet dynamics [120] fully accounts for all quantum-mechanical effects, and combined with high-quality potential energy surfaces, it can deliver highly accurate results. Regrettably, this method comes with a high price because it requires an effort that grows exponentially with the number of degrees of freedom. As a result, studies employing wave packet dynamics in nucleobases have been performed only in reduced dimensional models [121]. These scaling properties also extend to the closely related multi-configurational time-dependent Hartree (MCTDH) method [120], which employs a more compact, time-dependent basis set. This fully quantum-mechanical method has been employed for the description of a single nucleobase, for example as reported in [122], but for larger systems, MCTDH or its variants, like multilayer MCTDH [123], could only be used in an approximate manner by using restricted basis sets.

A powerful alternative to grid-based quantum dynamics methods is provided by Gaussian wave packet methods, where the wave packet is a linear combination of Gaussian functions [124]. These methods are still fully quantum-mechanical and formally converge to the exact solution of the time-dependent Schrödinger equation if the Gaussian basis fully covers the available phase space of the system. However, each Gaussian basis function is relatively localized, which allows the simulations to be run on-the-fly, where the information on the potential energy surfaces is only computed at the center of the Gaussian, thereby avoiding the exponential scaling of grid-based quantum dynamics. Examples of on-the-fly Gaussian-based quantum dynamics methods are direct-dynamics variational multi-configurational Gaussian [125], ab initio multiple spawning [126] and multiple cloning [127]. The main bottleneck in this type of simulation is given by the number of Gaussian basis functions, which depends strongly on the nature of the studied processes. Nonetheless, much larger molecule sizes than with grid-based dynamics should be accessible with Gaussian-based methods, and full-dimensional simulations of nucleobases can routinely be carried out, as has been done, e.g., for isolated thymine [52,128].

In the case of very large molecular systems, like long, fully-solvated DNA strands, even Gaussian-based quantum dynamics can become intractable, and one has to resort to a classical description of nuclear motion [129]. By treating the nuclei as point-like masses following Newton’s equation of motion, the effort for the dynamics simulation is reduced to linear scaling (although the effort to compute potential energies and gradients can still scale worse than linear). Excited-state dynamics can be treated using extensions of classical dynamics, with the most prominent method being surface hopping [130]. The applicability of classical dynamics to large systems comes with the high price of losing quantum-mechanical effects. If those turn out to be essential for the simulated process, one has to resort to an approximate treatment; see, e.g., [131]. Despite its limitations, there is a large amount of problems where quantum effects are not in the forefront, and surface hopping is able to deliver a qualitative correct insight into the dynamics of the system. Therefore, it is not surprising to find that surface hopping is by far the most popular dynamics method employed to date in the simulation of DNA photophysics, with applications ranging from isolated nucleobases to large systems, including the environment [6,74].

Another challenging task that arises when simulating large systems is the proper sampling of the configurational space. Large biomolecules can adopt conformations that are separated in configurational space by relatively large energy barriers, and thus, enhanced-sampling techniques are necessary to visit all relevant regions of the potential energy surface. This is an especially arduous problem for electronic excited states, where only picosecond time scale simulations can be evolved, even with a classical treatment of the nuclei. In the last few decades, a large amount of enhanced-sampling methods have been developed for the electronic ground state [132]. Likely, the most popular ones are those that use bias potentials to reduce the size of energy barriers allowing a fast minima-to-minima transition, such as umbrella sampling, metadynamics and hyperdynamics.[132]. These techniques can be directly applied to excited-state dynamics within the Born–Oppenheimer framework as was recently done for the simulation of thymine dimerization in the S${}_{1}$ state.[84]. However, the use of enhanced-sampling techniques in nonadiabatic simulations is much more involved because the bias of a potential energy surface would modify the energy gap between the states, artificially influencing the transition probabilities.

There exists a plethora of other methods for excited-state dynamics, which describe quantum effects to different extent, e.g., exact factorization [133], path integral methods [134,135,136] or Ehrenfest dynamics [137], and various others; see, e.g., [111,120,138,139]. Nonetheless, with all of these methods remains the challenge to balance the description of nuclear quantum effects with the feasibility of the computations, and thus, applications to DNA excited state dynamics are scarce [39].

Besides the choice of the dynamics method itself—quantum or classical—it is very important to consider the Hamiltonian terms, which couple the different electronic states. Nonadiabatic couplings are essential and can either be computed in the form of coupling vectors [140] or wavefunction overlaps [141,142]. Alternatively, diabatic coupling elements can be obtained through fits of the potential energy surfaces [29,143]. Depending on the process to simulate, also other couplings, like the interactions of the system’s dipole moment with an external electromagnetic field or the inclusion of relativistic effects, like spin-orbit coupling, need to be considered in the Hamiltonian. In this context, the surface hopping including arbitrary couplings (SHARC) method developed by us [113,144] is useful, and it has been extensively used to investigate the intersystem crossing in nucleobases [10,55,56,57,145] and nucleobase analogues [8,9,62].

## 5. Probe Processes

The advantage of numerical simulations is that they allow describing directly the excited-state dynamics as it happens on the actual potential surfaces. In the experiment, in contrast, the information about the dynamics is obtained only indirectly, since a so-called probe is necessary. This is a component that is important to be aware of, since the simulation of the excitation dynamics does not need to be directly comparable with the dynamics seen through the above-mentioned figurative lens, i.e., the probe dynamics. In order to avoid a possible comparison of apples with oranges, the simulations should ideally also include the detection process, as employed in the corresponding experiment. Comparing the very same observable allows detecting cases where the actual excited-state dynamics is encrypted and distorted in the experimental probe signal [22].

One prominent method applied in experiments on DNA’s light-induced dynamics is transient absorption spectroscopy, where the initially photo-excited molecule absorbs further light, and the absorption spectrum of this second excitation is recorded [3,74,146,147,148,149,150]. In order to simulate transient absorption spectra, one needs to include additional excited states beyond the ones involved in the dynamics itself and incorporate all of the corresponding transition dipole moments in the dynamics calculations. Then, one obtains oscillator strengths between the excited states populated in the dynamics and the higher-lying states accessible by the probe laser. Such calculations have been done at the stationary level for purine bases [8] and nucleobase analogs [9], as they are in principle straightforward. Methodologically, to include this probe only requires increasing the number of excited states in the calculations; but unfortunately, for many quantum chemical methods, the computational accuracy deteriorates with higher-lying excited states, and a meaningful transient absorption spectrum can be difficult to simulate. More rigorous methods to compute transient absorption spectra are also available [151].

Another experimental technique widely used to probe excited-state dynamics of the DNA’s constituents is photoelectron spectroscopy [74,152,153]. Here, the light used for the probing process contains more energy than in the case of transient absorption spectroscopy and is able to detach an electron from the molecule under study. Different aspects of this ionization process can be analyzed: one possibility is to merely count the number of ejected electrons; another possibility is to measure the velocity of these electrons or even to angularly resolve these velocities [154]. In general, two different types of ionization can be distinguished: single-photon ionization occurs at rather low laser intensities and short wavelengths. In contrast, multiphoton ionization is effective at high intensities and comparably long wavelengths. In order to simulate all of these processes, various approaches exist. It is generally easier to simulate single-photon ionization. In this case, a simple calculation of the ionization potential, i.e., the energy difference between the occupied excited state of the neutral molecule and the electronic ground state of the ionic molecule reached by the ionization process, might already provide a first insight. The calculation of Dyson norms offers an improvement at relatively low computational cost. Dyson norms are based on the overlap of the neutral and ionic wavefunctions, but neglect the outgoing electron [155]. Despite their simplicity, they allow for the calculation of the same observable as in the experiment and assist their interpretation, as in the dynamics simulations of thymine and uracil [52], cytosine [22,156], adenine [157] and nucleobase analogs [158]. The next level of amelioration is to incorporate the ejected electron by plane waves or Coulomb waves [159,160]. The kinetic energy of the outgoing electron can also be modeled by discretization of the ionic continuum [20,161,162,163,164]. Other approaches are based on b-splines [165] and Stieltjes imaging [166,167,168,169], where the electronic basis set is extended in different manners to describe the ejected electron. A better description needs not only to account better for the electronic continuum, but also to calculate transition dipole moments instead of mere overlaps. In this case, orientational averaging has to include the projection of the dipole moment vector on the laser polarization vector. In the involved calculations, the wavefunctions for neutral and ionic molecule, as well as the outgoing electron are coupled [170,171,172]. Such simulations are usually too expensive for time-resolved spectra, where several thousands of calculations are needed, but advantageously, they might be able to describe multiphoton ionization. In general, multiphoton ionization calculations need to involve many high-lying excited states, which are then Stark-shifted in the strong laser field, such that tiny inaccuracies in the state energies and the transition dipole moments can have huge effects [173]. That is the reason why they can be a real challenge if more than a qualitative picture is desired.

Other experimental approaches to map excited-state dynamics include 2D spectroscopy [174], high-harmonics generation [175], excited-state infrared spectroscopy [176], resonance Raman spectroscopy [177], circular dichroism [178] or 4D electron microscopy [179], among others. Most of these techniques have the problem that they aim at measuring a time-dependent quantity (e.g., the evolution of the excited-state populations) by employing another quantity with a possibly different time-dependence (e.g., the transition dipole moment changing along the different molecular geometries visited during the dynamics). Therefore, the inclusion of the detection process in the simulations is indispensable in order to get an adequate interpretation of the dynamics.

## 6. Analysis of the Results

The power of a computation does not only lie in reproducing experimental data, but in producing detailed information about the involved processes, which is difficult or impossible to get by the experiment alone. However, computations provide huge amounts of raw data, and sophisticated analysis techniques are required to reveal the most meaningful information about the structural motions and electronic wavefunctions driving the dynamics.

After absorption of light, individual nucleobases undergo structural modifications throughout the different excited-state pathways, which can be analyzed by monitoring the variation of internal coordinates (bond distances and angles, dihedral angles, out-of-plane motions, etc.) along the dynamics or stationary minimum-energy paths [62]. However, structural alterations do not occur only at the monomeric level, but also in the double-strand macromolecule. These changes are expected to be especially relevant when photoexcitation is followed by a chemical reaction, such as proton transfer between base pairs [46], proton transfer to the solvent [180], the dimerization of neighboring nucleobases [10], the binding of external molecules to the double strand [181], and others. Unfortunately, structural modifications of the DNA helix accompanying these photoinduced reactive events have been barely analyzed because the nucleic-acid environment is often absent in the simulations, and when it is included, a rigorous conformational analysis is often not performed. Admittedly, the conformational analysis of DNA structures is not straightforward because it depends on the reference system employed for the definition of structural variables. The two most widely-used approaches define the overall structure in terms of a helical axis [182] and in terms of parameters that define the link between successive base pairs [183]. Both methods are able to provide information about the overall curvature of the double strand, relative orientation between stacked and pairing nucleobases, shape and size of the minor and major grooves and conformations of the backbone. These types of tools are usually applied to classical ground-state molecular dynamics simulations [184,185], but they are hardly employed for the analysis of excited-state simulations.

Additional ways to explore geometrical variations, going beyond the traditional visualization of molecular internal coordinates, are normal mode analysis [186,187], essential dynamics analysis [30], Cremer–Pople parameters for ring geometries [188] and Boeyens classification for six-membered rings [189]. The post-processing of dynamics simulations or stationary pathways with the above-mentioned techniques can provide a complete picture of the conformational changes induced by light, not only in the individual nucleobases, but also in the entire macromolecule.

In addition to structural modifications, irradiation with light also induces changes in the electronic structure of DNA fragments. The most straightforward approach to analyze the electronic structure from excited state computations is to evaluate the response vector, consider one or a few leading electronic configurations, and visualize the orbitals involved. Such an approach works well for small systems with one or very few configurations, but can run into two major problems for general applications. First, it is problematic if the orbitals possess a mixed character, and consequently, the state character cannot be assigned without ambiguity. Second, this approach requires manual inspection for every individual state, which becomes cumbersome if a large number of computations are performed, e.g., in the case of dynamics simulations. The first problem can be solved with some well-established transformation techniques, such as the natural transition orbitals [190] and the attachment-detachment densities [191]. Solving the second problem requires an automated analysis of excited state character, which is a somewhat more involved task. In this context, an analysis strategy based on the one-electron transition density matrix was developed within the framework of exciton theory [27,192,193]. The central concept in this formalism is the computation of charge transfer numbers (cf. [194]), which partition the excited state into individual local and charge transfer contributions. These methods were initially applied by some of us to perform a decomposition of the absorption spectrum of nucleobase stacks into local, excitonic and charge transfer contributions [48], to characterize the wavefunctions in an exciplex formed between two adenine molecules [69] and to monitor excitation energy transfer in dynamics simulations of a DNA model system [195]. Other groups have subsequently used the formalism to analyze the effects of different stacking interactions [17,49], and the methodology could be generalized for the computation of excitonic coupling elements [60,196]. Related methods were applied in the analysis of double excitations relevant to thymine dimer formation [10] and to compare the excitations of nucleobases and their thio-substituted analogues [9].

For more general future applications, it will be beneficial to develop a formalism that does not only allow discriminating between different types of intermolecular excitations, as discussed above, but also intramolecular state characters. In this case, a proof-of-principle application has been presented for cytosine showing the general feasibility of differentiating between $\pi \pi^{*}$, $n\pi^{*}$ and Rydberg states based on numerical descriptors alone [197], but no general approach has been presented so far.

For the analysis of dynamics simulations, it is necessary to analyze the evolution of the electronic wavefunction in a meaningful way. In trajectory-based methods, the easiest way to do so is to track the populations of the adiabatic states ($S_{0},S_{1},T_{1},\ldots$); see, e.g., [55,66]. These populations can subsequently be fitted with simple exponential functions or subjected to more elaborate kinetic reaction modeling [57,62] to extract excited-state lifetimes, allowing for a comparison with experimental results. However, in many cases one would like to analyze the excited-state populations in terms of the diabatic state characters, since such a diabatic picture is often employed in the interpretation of experimental results. Changing from the adiabatic to the diabatic representation, where the kinetic coupling vectors are made zero, is not possible in a rigorous way since it would require considering an infinite number of states [111,198]. Practical approaches therefore involve regularization diabatization [199], on-the-fly diabatization [29,200] or approximate classifications, e.g., based on transition dipole moment strengths [145]. For a detailed study of the state characters, it is necessary to carry out a wavefunction analysis over the whole set of trajectories and present the results in a compact way. The feasibility of this idea has been illustrated in applications for energy and charge transfer model systems [195,201], but applications to DNA dynamics have not been reported so far. It will be particularly interesting to apply this procedure in the comparison of adiabatic surface hops (e.g., a transition from $S_{2}$ to $S_{1}$) and diabatic state changes (e.g., between a $\pi \pi^{*}$ and $n\pi^{*}$ state), in order to provide a unified description of excited state processes.

## 7. Conclusions

In this contribution, we present the particular view of the authors about which are the most important challenges associated with the simulation of photoinduced phenomena in DNA. We decompose a calculation of the processes occurring in an experiment into the following ingredients: (i) electronic structure theory; (ii) simulation of the initial excitation process; (iii) excited-state dynamics; (iv) calculation of probe processes; and (v) analysis of the results. In many studies, only a subset of these components is tackled, and the employed methods for these tasks have room for improvement. Popular techniques for these independent approaches are, e.g., stationary TDDFT calculations (sometimes in a QM/MM setup with electrostatic embedding), Δ-pulse excitations, mixed quantum-classical surface-hopping molecular dynamics, the omission of experimental probe processes and simple analysis of only the adiabatic state populations. Replacing these standard techniques by a full quantum-mechanical description without approximations for all ingredients will remain a utopia for quite a long time, but improvements on each of the independent tasks exist already today. In many cases, these techniques are more expensive and far from being black-box methods, restricting their widespread use. Since every technique offers specific advantages and disadvantages, no advice on routine procedures for simulations of light-induced processes can be given. The diversity of challenges for the different components calls for a community effort instead of idiosyncratic competition. Only a combination of experimental and computational expertise can help to put the pieces of the big puzzle together. Besides exploiting the currently-available possibilities, the development of new methods is of the utmost importance, because we are still far from realistic simulations of complete DNA double strands in their biological environment. Such developments also imply that questions already studied in the past might have to be critically scrutinized employing new techniques, and many fascinating phenomena are yet to be discovered.

## Abbreviations

The following abbreviations are used in this manuscript:

MDPI	Multidisciplinary Digital Publishing Institute
DOAJ	directory of open access journals
DNA	deoxyribonucleic acid
UV	ultraviolet
DFT	density functional theory
DFTB	density functional tight binding
TDDFT	time-dependent density functional theory
CC2	approximate coupled cluster
ADC	algebraic diagrammatic construction
CIS(D)	perturbatively-corrected configuration interaction with single excitations
CASSCF	complete active space self-consistent field
CASPT2	complete active space perturbation theory of second order
MRCI	multireference configuration interaction
QM	quantum mechanics
MM	molecular mechanics
DMRG	density matrix renormalization group
FCIQMC	full configuration interaction quantum Monte Carlo
MCTDH	multi-configurational time-dependent Hartree
SHARC	surface hopping including arbitrary couplings
